# Urbanisation threats to dairy cattle health: Insights from Greater Bengaluru, India

**DOI:** 10.1007/s11250-023-03737-7

**Published:** 2023-10-05

**Authors:** Md Shahin Alam, Silpa Mullakkalparambil Velayudhan, Debpriyo Kumar Dey, Chiamaka Adilieme, Pradeep Kumar Malik, Raghavendra Bhatta, Sven König, Eva Schlecht

**Affiliations:** 1 Animal Husbandry in the Tropics and Subtropics, University of Kassel and Georg-August-Universität Göttingen, Steinstraße 19, 37213 Witzenhausen, Germany; 2 Institute of Animal Breeding and Genetics, University of Gießen, Ludwigstraße 21B, 35390 Gießen, Germany; 3 ICAR-National Institute of Animal Nutrition and Physiology (NIANP), Hosur Road, Adugodi, Bengaluru, Karnataka 560030 India

**Keywords:** Cattle health, Dairy production, Food leftovers, Heavy metals, Lake fodder, Logit model, Megacity

## Abstract

Complex urbanisation dynamics, on the one hand, create a high demand for animal products, and on the other hand put enormous pressure on arable land with negative consequences for animal feed production. To explore the impact of accelerated urbanisation on dairy cattle health in urban farming systems, 151 farmers from different parts of the Greater Bengaluru metropolitan area in India were individually interviewed on aspects addressing cattle management and cattle health. In addition, 97 samples of forages from the shores of 10 different lakes, and vegetable leftovers used in cattle feeding were collected for nutritional analysis. Along with the use of cultivated forages, crop residues, and concentrate feed, 47% and 77% of the farmers occasionally or frequently used lake fodder and food leftovers, respectively. Nutritionally, lake fodder corresponded to high-quality pasture vegetation, but 43% of the samples contained toxic heavy metals such as arsenic, cadmium, chromium, and lead above official critical threshold levels. Therefore, lake fodder may affect cows’ health if consumed regularly; however, heavy metal concentrations varied between lakes (*P* < 0.05), but not between fodder types (*P* > 0.05). Although 60% of the interviewed farmers believed that their cows were in good health, logit model applications revealed that insufficient drinking water supply and the use of lake fodder negatively impacted cattle health (*P* < 0.05). While it remains unknown if regular feeding of lake fodder results in heavy metal accumulation in animal products, farmers and farm advisors must address this and other urbanization-related challenges to protect cattle health.

**Graphical abstract:**

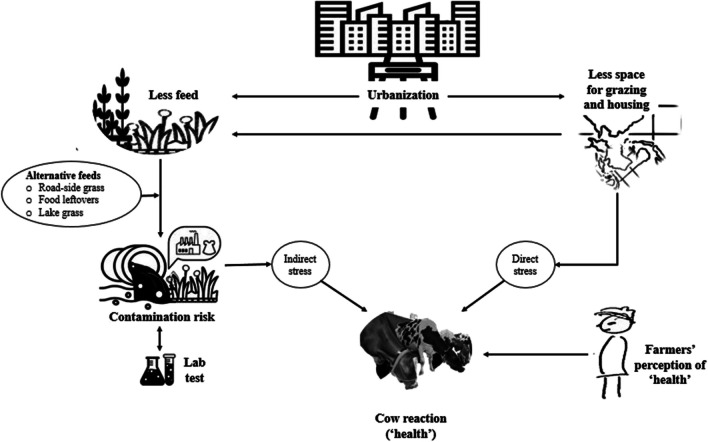

**Supplementary Information:**

The online version contains supplementary material available at 10.1007/s11250-023-03737-7.

## Introduction

**Fig. 1:**
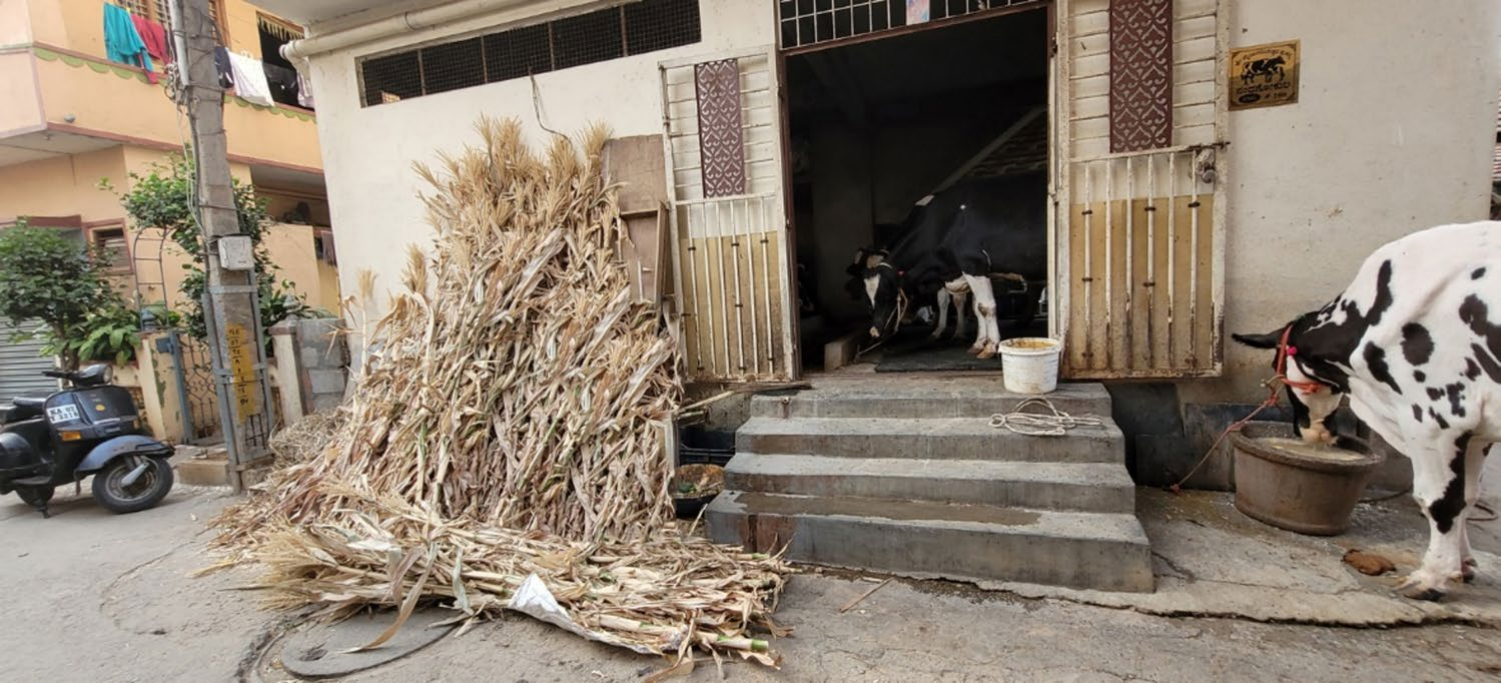
Cows kept in the basement of a house with sub-optimal space and ventilation facilities in an urban neighborhood of Greater Bengaluru, India

Maintaining animal health is a key concern for all livestock farmers, and is integral to sustainable livestock farming (Tlusty 2020). However, complex urbanisation dynamics, on the one hand, create a high demand for animal products (Steinfeld et al. 2006; Thornton 2010), and on the other hand, put enormous pressure on arable land for the construction of metropolitan infrastructure with negative consequences for animal feed production (Thornton 2010; Seto and Ramankutty 2016; Swain and Teufel 2017). Therefore, many livestock keepers in rapidly urbanising areas are dependent on dwindling common pool resources for feeding their animals (Seto and Ramankutty 2016; Mundoli et al. 2022) or increase the use of food leftovers to feed their animals (Prasad et al., 2019; Takiya et al., 2019; Reichenbach et al. 2021a). Simultaneously, land scarcity often entails poor housing conditions for animals, such as restraining cattle in the basement of houses or in an empty room with sub-optimal ventilation facilities (Fig. 1). As a result, many urban livestock keepers in so-called developing or transition countries tie up animals in open yards or on sidewalks of roads, or leave them scavenging in the streets during the daytime (Thuo 2013; Pinto et al. 2021). In consequence, prolonged exposure to the sun, particularly during summer, leads to heat stress (Lees et al. 2019; Velayudhan et al. 2022).

In the last decade, dairy farming systems rapidly changed in the megacity of Bengaluru, which is one of the fastest-growing urban agglomerations in southern India (Prasad et al. 2019; Reichenbach et al. 2021a). For roughage acquisition, most dairy farmers traditionally relied on grazing or cultivating fodder on their own land, but rapid urbanisation processes transform agricultural land into an urban fringe (Thornton 2010; Seto and Ramankutty 2016; Swain and Teufel 2017). As a consequence, dairy farmers are forced to use alternative feed resources (Prasad et al. 2019; Reichenbach et al. 2021a; Alam et al. 2022), for example vegetation from public lakeshores, parks, roadsides, and designated construction areas (Swain and Teufel 2017; Reichenbach et al. 2021a; Mundoli et al. 2022), as well as household vegetable and food waste, fruit and vegetable waste from wet markets, and industrial fruit peels (Prasad et al. 2019; Takiya et al. 2019; Reichenbach et al. 2021a). Although the use of food leftovers and publicly available feed resources is widely practiced in the Greater Bengaluru region (Reichenbach et al. 2021a), the nutritional quality of such feed resources has been neglected so far. What has, however, been reported in a few studies is that chromium (Cr), lead (Pb), and cadmium (Cd) concentrations are alarmingly high in soil, water, and vegetation from lakes in Bengaluru (Varalakshmi and Ganeshamurthy 2012; Ramachandra et al. 2018, 2020). For example, the ranges of Cr (34.2—166.4 mg kg^−1^ DM) and Pb (12.8—59.7 mg kg^−1^ DM) concentrations in unclassified macrophytes from Bellandur lake were twelve times higher than the WHO threshold values (Ramachandra et al. 2018), and similar results were reported for macrophytes from Varthur lake (Ramachandra et al. 2020). The intake of such edible macrophytes by cattle can affect their fertility by provoking abortions, premature calving, oocyte dysfunction, disruption of spermatogenesis, sperm apoptosis and oxidative damage, as well as the functioning of kidneys, the cardiovascular and nervous system (Dhaliwal and Sushma 2016; Alam and Silpa 2020; Guvvala et al. 2020; Volkov and Ezhkova 2020; Wrzecińska et al. 2021).

However, since urbanisation increases the market demand for animal source food such as milk, urban livestock farmers try to withstand the multiple challenges of the city environment and continue their activity by exploiting new opportunities, such as the increased availability of vegetable residues (Reichenbach et al. 2021b). Although their adaptive strategies may entail positive aspects for the urban environment, such as waste upcycling and reduced enteric methane emissions from dairy cattle (Pinto et al. 2020a; Reichenbach et al. 2021a), increased heat stress and impaired cattle health may be the cost to be borne by the animals (Pinto et al. 2020b; Velayudhan et al. 2023). While the latter authors have primarily investigated the impact of housing infrastructure on animal health indicators, the present study aimed to identify the health impacts arising from animal management and feeding of publicly available feed resources, especially lake fodder, in the highly dynamic context of Greater Bengaluru.

## Materials and methods

### Study location

**Fig. 2:**
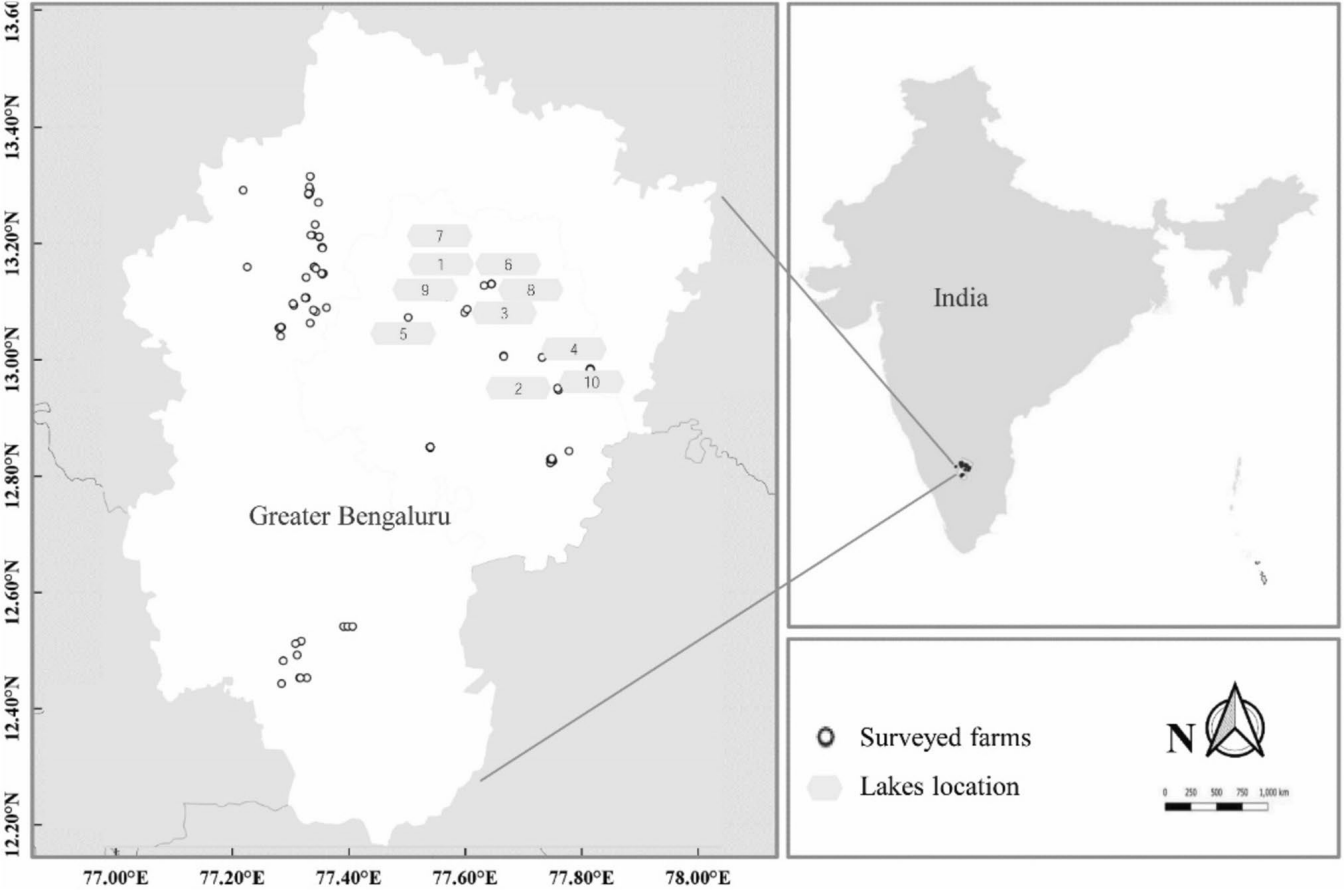
India (right), and the Greater Bengaluru region (left) with location of 151 surveyed dairy farms and 10 surveyed lakes. *Lake names: 1* = *Attur, 2* = *Bellandur, 3* = *Doddabomasandra, 4* = *Doddanekundi, 5* = *Narasappanahalli, 6* = *Puttenhalli, 7* = *Ramgondanhalli, 8* = *Sampegalli, 9* = *Singapura, 10* = *Varthur*

The study was conducted in the Greater Bengaluru region (Fig. 2). Bengaluru is the capital city of the southern Indian state of Karnataka and has about 13 million inhabitants at present (United Nations 2022). Located at an elevation of 600 to 900 m above sea level (a.s.l.) in the southern half and 300 to 600 m a.s.l. in the north on the Deccan plateau, ambient temperatures in Bengaluru averaged 26 °C across the year and yearly rainfall averaged 1146 mm in the period 1989—2018 (Guhathakurta et al. 2020; WWO 2020). Across the city’s urban, peri-urban, and rural areas, dairy farming is present and contributes to the income of the involved families (Chandrasekar et al. 2017). The region was reported to host approximately 137,000 cattle with a total milk production of about 295,000 L per year (NDDB 2015). If possible, dairy farmers grow African tall maize (*Zea mays* L.) and hybrid Napier grass (*Pennisetum purpureum* Schumach.) on their own land, and cultivate or buy finger millet straw (*Eleusine coracana* Gaertn.). Farmers unable to cultivate forage often harvest or allow their cows to graze alligator weed (*Alternanthera philoxeroides* (Mart.) Griseb.), Bermuda grass (*Cynodon dactylon* (L.) Pers.), para grass (*Brachiaria mutica* Stapf), water hyacinth (*Pontederia crassipes* Mart.), and vetiver (*Chrysopogon zizanioides* (L.) Roberty) on lake shores, and collect fruit and vegetable leftovers from neighbors, markets, restaurants, and food processing industries (Reichenbach et al. 2021a; Alam et al. 2022). The listed 210 lakes in Greater Bengaluru (KLCDA 2019) are important for replenishing the regional groundwater level, but are at the same time used for washing clothes, irrigating field crops, religious and recreation purposes, fishing, swimming, and cattle grazing (Mundoli et al. 2015).

### Selection of lakes and farms

During the period 1 December 2019 to 10 January 2020, different lakes in the Greater Bengaluru region were purposefully visited, based on a list of the Karnataka Lake Conservation and Development Authority (KLCDA 2019). Wherever farmers were observed to harvest grass or where cattle were grazing in the lake area, the farmer was approached directly or was identified via the animals, respectively. These initial farmers were then contacted and asked for the names of other farmers who were also using the lake’s fodder resources. In this way, 104 dairy farmers from the neighborhoods of 10 different lakes were identified who kept at least two lactating or dry cows or already inseminated heifers for market-oriented milk production. In addition, 47 dairy farmers who did not rely on forages from lake shores were randomly selected from the name list of a previous survey (Pinto et al. 2020b). All 151 farmers were approached for an interview; prior to that, the purpose of the survey was explained to them and their oral consent was requested. Only if a farmer agreed to partake in the survey, she / he was interviewed in a face-to-face conversation at the farm using a structured questionnaire with open and closed questions. Interviews were carried out between 18 January and 20 March 2020. Each farm location was georeferenced with a handheld GPS device (Garmin eTrex 10, Schaffhausen, Switzerland). Interviews explored socio-economic aspects of the farm, cattle feed origin, feed availability, way of feed acquisition, cattle feeding and watering strategies, animal housing and manure management, as well as the farmer’s appraisal of the health of her / his cattle (Appendix I). Furthermore, milk production, milk marketing, use of farming inputs, advisory services, and long-term farm management plans were addressed. Conduction of the survey had received ethical approval by the Institutional Animal Ethics Committee (IAEC), National Institute of Animal Nutrition and Physiology (NIANP), Bengaluru, India (NIANP/IAEC/1/2020/6). Permission for vegetation sampling on lake shores (see 2.3) had been granted by the Government of Karnataka (APAJEE7EPC2021).

### Lake fodder sampling

In May 2022, 92 samples of the different types of fodder collected by farmers for feeding their animals or grazed by cattle were collected from the 10 lakes used by the surveyed dairy farmers (Fig. 1). Samples included alligator weed (*n* = 26), Bermuda grass (*n* = 5), para grass (*n* = 14), water hyacinth (*n* = 17), and mixes of species (Bermuda grass, para grass, water hyacinth plus alligator weed; *n* = 30); plant species were identified based on standard taxonomic literature (Cook 1996). Additionally, five samples of food leftovers (household food waste, wet market vegetable waste, restaurant food waste, industrial fruit peels) were collected on five surveyed farms. The time delay between conduction of the interviews and collection of the samples was due to the COVID-19 restrictions in India that were imposed shortly after the end of the survey (March 2020). Upon collection, vegetation samples were stored in micro-perforated polythene bags. At the premises of the Dry Land Agriculture Laboratory at the University of Agricultural Sciences Bangalore, the samples were weighed (precision: 0.01 g, KERN ew, Munich, Germany), pre-dried for three days in a solar dryer consisting of wooden frames covered with UV-stable polyethylene sheets, and then dried in a forced convection oven at 60 °C (Equitron-stream series No. 7051–250, Mumbai, India) for 2 to 3 days until constant weight was reached. Immediately after drying, the samples were reweighed, ground in a mixer grinder and sieved to pass a 0.5 mm mesh. Ground samples were stored air-dry in plastic zip-lock bags until analysis.

### Feed quality analysis

Dry matter (DM) and ash concentrations in samples of lake fodder and food leftovers were determined according to the German Handbook of Agricultural Research and Analytic Methods (VDLUFA 2012; method 3.1 and 8.1, respectively). Organic matter was calculated by subtracting the concentration of ash from DM set at 100% (VDLUFA 2012). The nitrogen (N) concentration was determined with a Vario Max C/N analyzer (Elementar Analysensysteme GmbH, Langenselbold, Germany) and crude protein (CP) was calculated by multiplying N concentration with the factor 6.25. Calcium (Ca) was determined using a BWB-XP flame photometer (BWB Technologies, UK Ltd.); the procedure involved mixing 0.5 ml of sample solution, 0.3 ml of Lathan (III)-oxide and 9.2 ml of deionised water, thereby considering the dilution factor during analysis (Puffeles and Nessim 1959). Phosphorus (P) concentration was determined by spectrophotometry (Hitachi U-2000, Tokyo, Japan) at an absorption wavelength of 460 nm using the vanadate-molybdate method (Gericke and Kurmies 1952). The neutral detergent fibre (NDF) and acid detergent fibre (ADF) fractions were determined sequentially in an Ankom^200^ Fiber analyzer (ANKOM Technology, NY, USA) with filter bags F57 and following the operator’s manual (https://www.ankom.com/sites/default/files/document-files/A200_Manual.pdf).

For the analysis of heavy metal concentrations, the samples underwent a microwave-assisted digestion (Anton Paar, Graz, Austria). Approximately 0.5 g fully dried and grinded sample material was placed in a marked polytetrafluoroethylene microwave digestion tube. A volume of 6 ml conc. HNO_3_ (Supra 69%, Roth, Germany) was added and the vessel was placed in the microwave digester. Then the sample was pre-heated (100 °C for 10 min, holding time 5 min), heated (180 °C for 10 min, holding time 5 min), digested (190 °C for 5 min, holding time 15 min), and cooled (55 °C for 23 min). After acid digestion, 1 ml of HCl (Supra 30%, Roth, Germany) was added to each vessel, along with demineralized water to complete a volume of 50 ml. Afterwards, the thus treated sample solids were recovered by filtering (Whatman No.40 paper) and preserved in a polyethylene bottle for further analysis (Paar 2020). A reagent blank sample was also prepared for each batch.

Concentrations of arsenic (As), Cd, Cr, and Pb were determined by inductively coupled plasma - optical emission spectroscopy (ICP-OES) using a Spectrogreen ICP-OES analyzer (SPECTRO, Kleve, Germany) equipped with SPECTRO ICP Analyzer Pro software. Argon was used as plasma gas. Calibration standards were prepared by serial dilution using a dilute nitric acid and hydrochloric acid-matrix based aqueous solution of 100 mg l^−1^ (ppm) (Supelco, Centipur® ICP multi-element standard solution IV, Cat. No. 1.11355.0100, Merck, Germany). The detection limits were as follows: As = 0.006093 mg l^−1^, Cd = 0.000130 mg l^−1^, Cr = 0.000752 mg l^−1^, and Pb = 0.003022 mg l^−1^. Three technical replicates were analysed for all samples. For Cd, Cr, and Pb, the accuracy of the analytical procedure was tested by analyzing a reference material (Rye grass, ERM®-CD281, Geel, Belgium) together with the forage samples. Obtained concentrations (Cd = 0.126 mg kg^−1^ DM, Cr = 23.6 mg kg^−1^ DM, Pb = 1.77 mg kg^−1^ DM) were within in the certified range of the reference material (Cd = 0.120 ± 0.007 mg kg^−1^ DM, Cr = 24.3 ± 1.3 mg kg^−1^ DM, Pb = 1.67 ± 0.11 mg kg^−1^ DM). The analytical method was tested by analyzing blank samples (*n* = 3) and no major interferences were found in the quantitative element analysis. The results were also validated by inter-laboratory comparison of 10% of the samples with analysis by AGROLAB LUFA GmbH (Kiel, Germany); differences in concentrations of As, Cd, Cr, and Pb were insignificant (*P* < 0.05).

### Data analysis

Statistical analyses were performed in R (version 4.0.3). Descriptive statistics (percentage, mean, standard deviation, median) were calculated for cattle management and health variables (survey) as well as for plant sample nutrient and heavy metal concentrations using the *tidyverse* function in base R (Wickham et al. 2019). A binomial test was performed on heavy metal concentrations for detected and not-detected levels. Normality was tested separately for each element by visually assessing individual Q-Q plots; samples’ equal variances were checked separately for each element with Levene’s test (O’Neill and Mathews 2000). As all variables other than Ca and As were normally distributed and showed homogeneity of variance, one-way analysis of variance (ANOVA) was computed. In case of significant differences (*P* < 0.05) in the global test, the Šidák post-hoc test was computed for pairwise comparisons among the feed types and lakes (Midway et al. 2020) using the *multcomp* function in R (Hothorn et al. 2016). For the non-normally distributed concentrations of Ca and As, the Kruskal Wallis test was performed and in case of significant differences (*P* < 0.05) in the global test, Dunn’s post-hoc test was computed for pairwise comparison among the feed types and lakes (Midway et al. 2020).

**Fig. 3:**
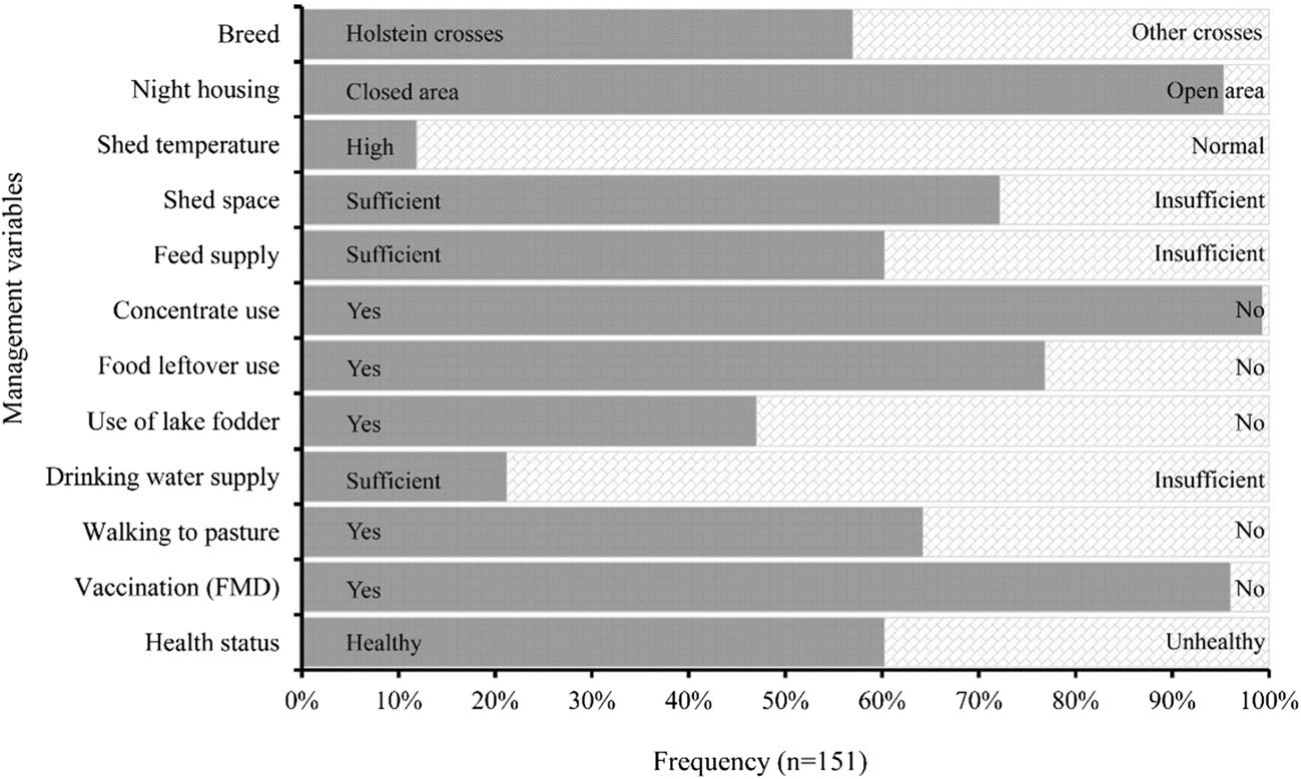
Frequency distribution of key herd management variables and of perceived cattle health (bottom bar: health status) across 151 surveyed dairy farms in Greater Bengaluru, India. *Holstein crosses: Crosses of Holstein Friesian with local cattle breeds*. *Other crosses: Crosses of Jersey, Sindhi and Sahiwal with local cattle of undefined genetics and pure local breeds such as Amritmahal and Gir*. *FMD: Foot-and-mouth disease*

Regression analysis was used to predict farmers’ appreciation of cattle health status at farm level from a set of independent variables (x). The dependent variable, y, was dichotomous and took the value 1 for the probability of healthy cattle and the value 0 for the probability of unhealthy cattle. Similarly, all independent variables (breed, night housing, shed temperature, shed space, feed supply, concentrate use, food leftover use, use of lake fodder, drinking water supply, walking to pasture, and vaccination) were dichotomous (Fig. 3), and the relationship between the dependent and independent variables was a non-linear function. Therefore, the logistic regression function was used, that is the logit transformation of y (Gujarati 2003):

$$\mathrm{Logit}\left[\mathrm{y}\left(\mathrm{x}\right)\right]= \alpha +{\beta }_{1}{x}_{1}+{\beta }_{2}{x}_{2}\dots .+{\beta }_{i}{x}_{i}$$

where

α: equation constant
*β*_*1 to i*_: coefficients of the independent variables

A functional test for multi-collinearity was performed for all independent variables obtained from the survey before running the logit model. The pairwise correlation between independent variables was computed in a model matrix arrangement (O’Grady and Medoff 1988) using the *ggcorrplot* function in R (Kassambara 2022). The correlation values (R) were found ≤ 0.36 for the independent variables, confirming that none of them were highly associated, and therefore all were included in the logit model (Mukaka 2012). In the output of the logit model, the positive or negative sign of the *β* coefficient indicates the direction of the relationship between a given independent variable x and the dependent variable y, while the computed average marginal effects (AME) estimate indicates the magnitude of change in the dependent variable for a 1-unit alteration in the given independent variable. The following model was used to estimate the AME using the *margins* (Leeper 2021) function in R (Gujarati 2003; Uddin et al. 2019):

$$\mathrm{dZ}/\mathrm{dQ}={\beta }_{\mathrm{i}}\left\{{P}_{\mathrm{i}}\left(1-{P}_{\mathrm{i}}\right)\right\}$$

where dZ is the probability of healthy cattle, dQ is the probability of independent variables, *β*_*i*_ is the estimated logit regression coefficient with respect to the *i*^th^ determinant of cattle health, and *P*_*i*_ is the estimated probability of cattle health status. The model fit test was computed by goodness-of-fit (Chi square, and Cox and Snell R^2^), comparing maximum likelihood (Nagelkerke R^2^ and McFadden R^2^) and the values of AUC, which is the Area Under the ROC (Receiver Operating Characteristics) curve (Wu et al. 2015).

## Results

### Characteristics of dairy farms

The 151 surveyed dairy farmers kept between 2 and 10 heifers, dry, and lactating cattle (mean = 4.41 cows farm^−1^, SD = 2.24) plus 1 to 5 calves. The majority of cattle (58%) were crossbreds of Holstein Friesian with local cattle breeds (Holstein crosses); the remaining animals were crossbreeds (other crosses) of Jersey, Sindhi, and Sahiwal with local cattle, and pure local breeds such as Amritmahal and Gir (Fig. 3). The average milk yield per lactating cow was 8.4 l day^−1^ (SD = 4.36). The majority (78%) of dairy farmers sold the milk to the Karnataka Milk Federation (KMF) of which they were members; the remaining 22% of farmers sold the milk directly to private consumers and restaurants.

**Fig. 4:**
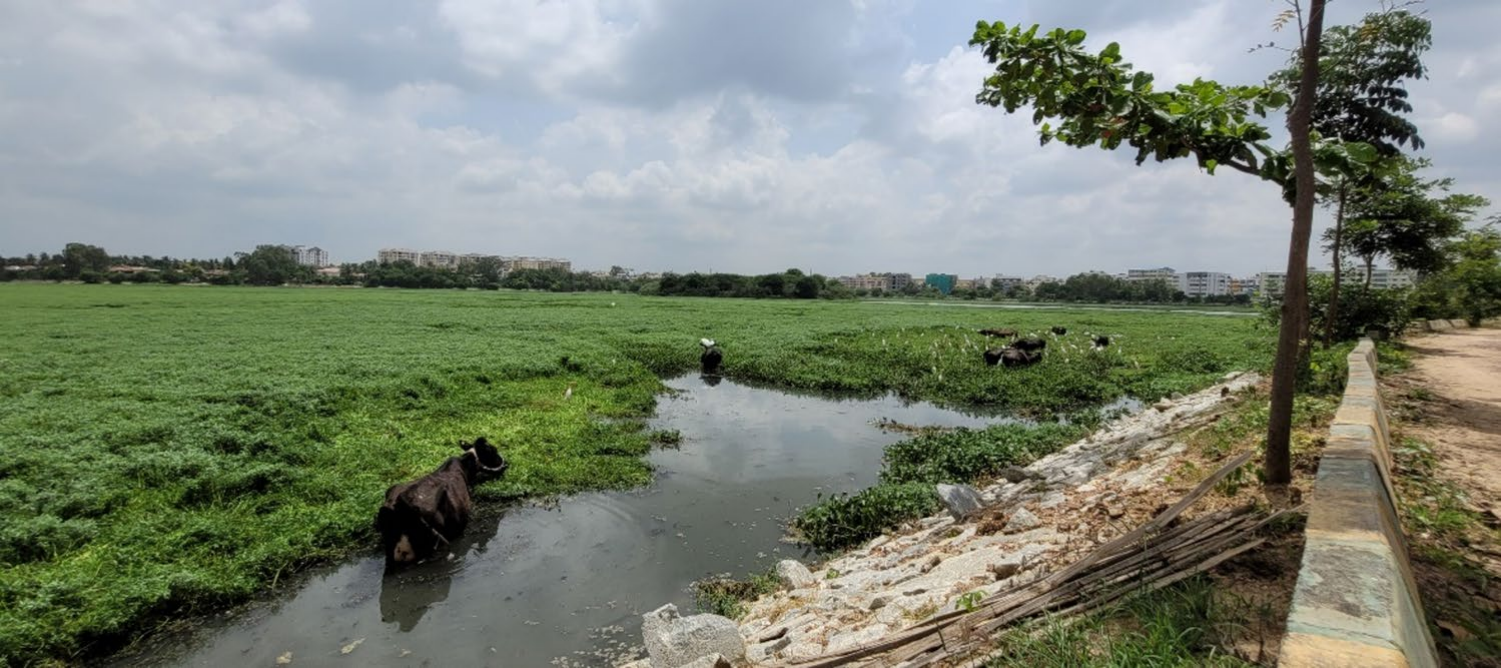
Cows grazing in Doddanekundi lake in the urban area of Greater Bengaluru, India

The majority (94%) of dairy farmers housed their cattle in a closed night shed, whereby 10% of the interviewees mentioned the problem of high temperatures in the shed. Most farmers offered concentrate feeds (99% of farmers) and food leftovers (77%) to their animals. Cattle were also fed with cultivated (African tall maize, hybrid Napier grass) or naturally growing (Bermuda grass, vetiver grass) green forages, as well as with cultivated or purchased dry forages, mainly consisting of finger millet straw. Of the interviewed dairy farmers, 47% regularly sent their cows for grazing near lakes, or harvested or purchased fodder plants growing on lake shores (Fig. 4); the average lake shore distance to the dairy farm was 2.11 km (SD = 2.01). Moreover, 63% of the dairy farmers sent their cattle out of the farm for walking and grazing on a daily basis.

Only 20% of the dairy farmers reported that they were able to supply sufficient drinking water to their cattle on a daily basis, while 95% vaccinated their cattle against FMD. Overall, most farmers were confident that their cattle were in good health (60%), were kept in sheds with adequate space (72%), and were supplied with sufficient amounts of feed (60%).

### Nutritional quality of lake fodder and food leftovers

The DM content of Bermuda grass differed (*P* < 0.05) from that of alligator weed, water hyacinth, and mixed grass species (Table 1). With values exceeding 150 g kg^−1^ DM in most cases, the crude ash content of the different fodder types was similar but high, indicating contamination with sand particles from lake sediments. Alligator weed had a higher concentration (*P* < 0.05) of CP than Bermuda grass and water hyacinth, whereas NDF and ADF concentrations were lower (*P* < 0.05) in alligator weed and food leftovers than in the other fodder types. The Ca and P concentrations were higher (*P* < 0.05) in food leftovers and water hyacinth than in the other fodder types.

**Table 1:** Average concentration of dry matter (DM), ash, crude protein (CP), neutral detergent fiber (NDF), acid detergent fiber (ADF), calcium (median), and phosphorus in different types of feed samples collected in Greater Bengaluru, India

Feed	Alligator weed (*n* = 27)	Bermuda grass (*n* = 5)	Para grass (*n* = 13)	Water hyacinth (*n* = 17)	Mixed grasses* (*n* = 30)	Food leftovers (*n* = 5)	Overall SEM
Nutrients							
DM (g kg^−1^ FM)	128.0^a^	177.5^b^	148.4^ab^	118.4^a^	133.3^a^	154.2^ab^	5.6
Ash (g kg^−1^ DM)	177.9	160.5	170.6	159.6	160.5	117.5	6.8
CP (g kg^−1^ DM)	229.3^b^	140.8^a^	182.8^ab^	154.3^a^	194.9^ab^	193.7^ab^	7.1
NDF (g kg^−1^ DM)	373.4^a^	563.6^b^	587.6^b^	573.9^b^	532.4^b^	268.4^a^	8.9
ADF (g kg^−1^ DM)	234.2^ab^	283.1^bcd^	328.1^d^	275.6^c^	286.4^c^	189.3^a^	6.6
Ca (g kg^−1^ DM)	16.4^a^	9.1^b^	8.3^b^	25.1^c^	11.7^b^	30.2^ac^	7.2
P (g kg^−1^ DM)	3.4^a^	3.1^ab^	3.1^a^	4.8^b^	4.0^ab^	5.1^b^	1.1

*SEM*: Standard error of mean and standard error of median (Ca), respectively; *FM*: Fresh matter; *DM*: Dry matter; *CP*: Crude protein; *NDF*: Neutral detergent fiber; *ADF*: Acid detergent fiber; * mix of Bermuda grass, alligator weed, para grass and water hyacinth. Values with different superscript letters within the same row differ at *P* < 0.05

The proportion of samples with detected concentrations of heavy metals and those below detection limit (Table 2) differed for each element (*P* < 0.05). Concentrations of the four selected heavy metals in different types of lake fodder ranged from 0.58—8.69 mg As kg^−1^ DM, 0.02—6.25 mg Cd kg^−1^ DM, 0.62—48.47 mg Cr kg^−1^ DM, and 0.48—29.99 mg Pb kg^−1^ DM (Table 2), with 43%, 14%, 92%, and 22% of the samples exceeding the respective official threshold concentrations for feedstuffs. In food leftovers, no traces were detected for As. However, Cd, Cr, and Pb ranged from 0.08—0.12, 1.81—5.44, and 0.57—3.14 mg kg^−1^ DM, respectively, with all samples exceeding the threshold for Cr.

**Table 2:** Heavy metal concentrations (mg kg^−1^ DM) in lake fodder (*n* = 92) and food leftovers (*n* = 5) used for feeding dairy cows in Greater Bengaluru, India

Heavy metal	Samples detected (%) *	Samples (%) below detection limit	Samples (%) above EU threshold	Lake fodder		Food leftovers		EU threshold**
				Mean ± SD	Median	Mean ± SD	Median	
As	55	45	43	2.54 ± 1.71	1.92	ND	ND	2.00
Cd	97	3	14	0.72 ± 1.79	0.18	0.10 ± 0.02	0.10	1.00
Cr	96	4	92	11.02 ± 15.71	5.79	3.47 ± 1.48	3.53	1.30***
Pb	98	2	22	3.99 ± 5.47	1.82	1.35 ± 1.21	0.85	5.00

*SD* = Standard deviation; *ND* = Not-detected; *Binomial test for all elements at detected and undetected level differed at *P* < 0.05; ** EU threshold (López-Alonso 2012); ***World Health Organization threshold (Ramachandra et al. 2018)

**Fig. 5:**
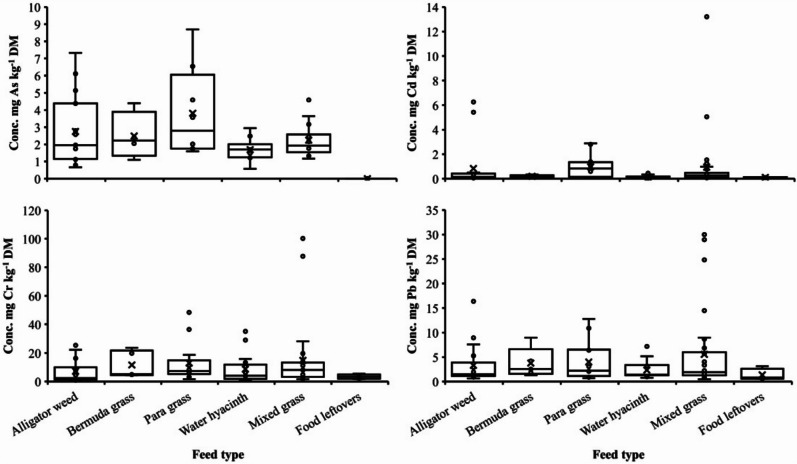
Concentration (Conc.) of the heavy metals arsenic (As, *n* = 48), cadmium (Cd, *n* = 81), chromium (Cr, *n* = 88) and lead (Pb, *n* = 80) in different lake fodder types and food leftovers collected in Greater Bengaluru, India. The horizontal bar in a box depicts the median, lower and upper limits of a box indicate the 25^th^ and 75^th^ percentile, vertical lines show the 95% data range and crosses depict mean values, while dots denote outlier values. Please note the different scales of the y-axes

Concentrations of Cr were highest in the different lake fodder types, followed by concentrations of Pb, As, and Cd (Fig. 5). Para grass showed higher average concentrations of As, Cd, Cr, and Pb (4.10, 1.05, 12.98, and 3.99 mg kg^−1^ DM, respectively) than the other fodder types, but there were no significant differences in heavy metal concentrations between fodder types.

**Fig. 6:**
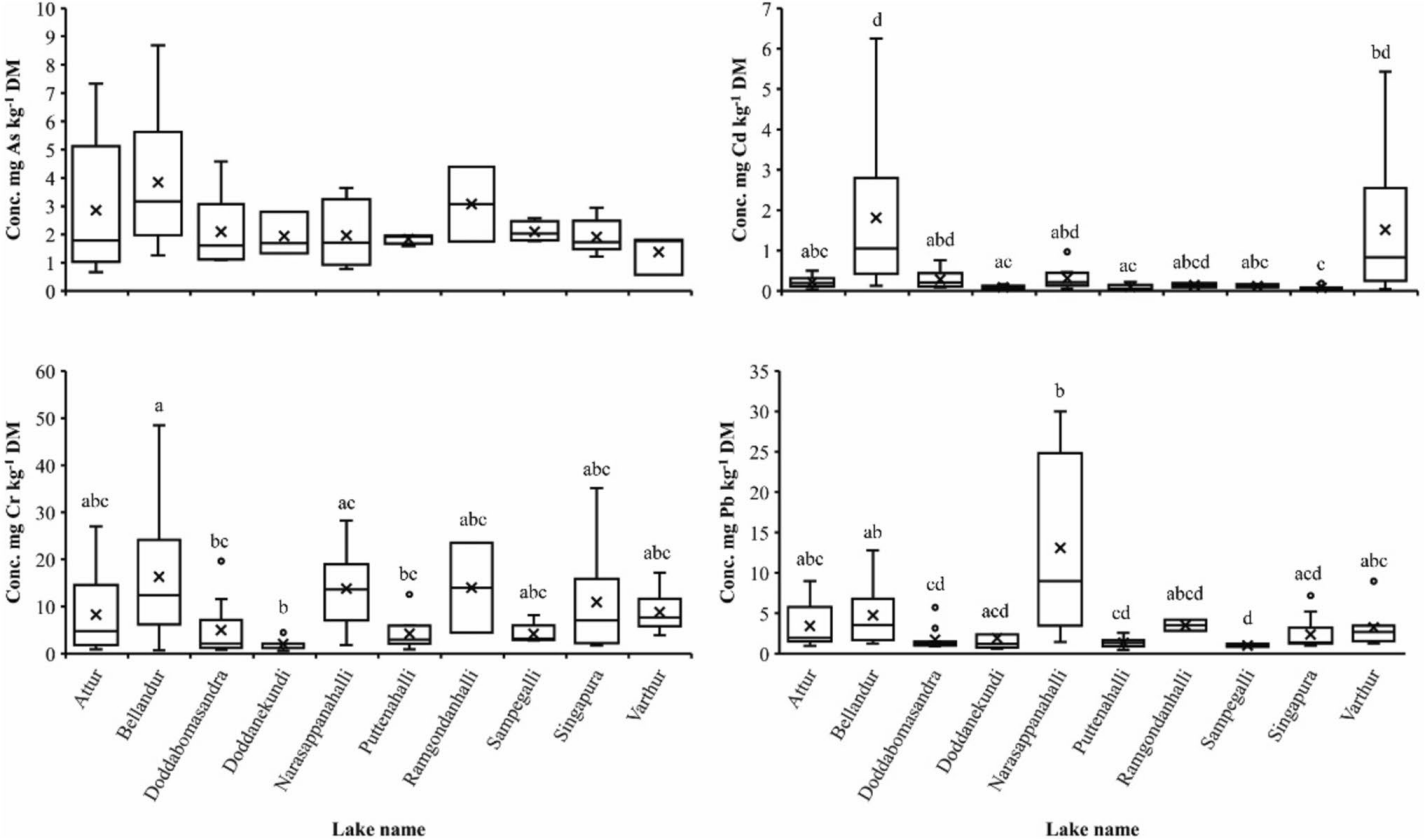
Concentration (Conc.) of the heavy metals arsenic (As, *n* = 48), cadmium (Cd, *n* = 76), chromium (Cr, *n* = 83) and lead (Pb, *n* = 78) in fodder growing on the shores of ten different lakes in Greater Bengaluru, India. The horizontal bar in a box depicts the median, lower and upper limits of a box indicate the 25^th^ and 75^th^ percentile, vertical lines show the 95% data range and crosses depict mean values, while dots denote outlier values. Please note the different scales of the y-axes. *Boxplots with different superscript letters differ at P* < *0.05*

The concentrations of Cd, Cr, and Pb, respectively, differed (*P* < 0.05) between the lakes, which was not the case for As (Fig. 6), although fodder from Bellandur lake had higher As concentrations than fodder from other lakes. The Cd concentration in fodder from Bellandur lake (1.81 mg Cd kg^−1^ DM) was higher (*P* < 0.05) than that in fodder from Attur (0.22 mg Cd kg^−1^ DM), Doddanekundi (0.09 mg Cd kg^−1^ DM), Puttenahalli (0.10 mg Cd kg^−1^ DM), Sampegalli (0.12 mg Cd kg^−1^ DM), and Singapura (0.08 mg Cd kg^−1^ DM) lakes. In case of Cr, fodder from Bellandur lake had again a higher (*P* < 0.05) concentration (16.32 mg Cr kg^−1^ DM) than fodder harvested from Doddabomsandra (5.01 mg Cr kg^−1^ DM), Doddanekundi (2.08 mg Cr kg^−1^ DM), and Puttenhalli (4.27 mg Cr kg^−1^ DM) lakes. In the case of Pb, fodder from Narasappanahalli lake had the highest (*P* < 0.05) concentration (13.09 mg Pb kg^−1^ DM), followed by Singapura (2.38 mg Pb kg^−1^ DM), Doddanekundi (1.93 mg Pb kg^−1^ DM), Doddabomsandra (1.73 mg Pb kg^−1^ DM), Puttenahalli (1.36 mg Pb kg^−1^ DM), and Sampegalli (1.02 mg Pb kg^−1^ DM) lakes.

### Factors influencing cattle health

The logit model tested the impact of farmers’ stated management practices on the perceived health of their dairy cattle herd. Results showed that a high share of (first generation) Holstein crosses, sufficient feed supply, use of concentrate feed, use of food leftovers, availability of a closed night shed, adequate shed space, daily walking to pasture, and FMD vaccination had a positive effect on cattle health (Table 3). Conversely, the use of lake fodder, insufficient supply of drinking water, and high shed temperature had a negative effect on cattle health.

**Table 3:** Coefficient estimate (β***i***), standard error (SE), average marginal effects (AME), and Z values of the logit model predicting the effect of management variables on cattle health as perceived by farmers

Variable	β***i***	SE	AME
Constant	-14.06	882.74	-
Dominant genetics: exotic crosses	0.94*	0.41	0.17
Use of closed night shed	0.62	0.94	0.11
High shed temperature	-0.67	0.60	-0.13
Adequate shed space	0.31	0.45	0.06
Sufficient feed supply	0.83*	0.42	0.15
Concentrate use	12.32	882.74	0.60
Food leftover use	0.37	0.45	0.06
Use of lake fodder	-1.49**	0.42	-0.29
Inadequate drinking water supply	-0.94*	0.47	-0.18
Walking to pasture	0.54	0.43	0.10
Vaccination (FMD)	0.79	1.02	0.15

*FMD*: Foot-and-mouth disease; * and ** indicate significance at *P* < 0.05 and *P* < 0.01, respectively

*Model fit results:*

*Model χ2* = *37.75, df* = *11, P* = *0.000*

*AUC (Area Under the Curve)* = *0.75*

*Cox and Snell R*^*2*^ = *0.22*

*Nagelkerke R*^*2*^ = *0.29*

*McFadden R*^*2*^ = *0.20*

Among the beneficial variables, especially Holstein genetics and sufficient feed supply had a significant positive effect (*P* < 0.05). In contrast, insufficient drinking water supply (*P* < 0.05) and use of lake fodder (*P* < 0.01) had a significant negative effect on cattle health. The result for the average marginal effect of using lake fodder (AME = -0.29) indicated that increasing the use of lake fodder has a stronger negative effect on cattle health than high shed temperature (AME = -0.13) and inadequate supply of drinking water (AME = -0.18).

## Discussion

The present analysis of small-scale dairy farmers’ coping strategies with respect to the multiple challenges posed to their activities by the rapidly progressing urbanisation in the megacity of Bengaluru provided a number of relevant insights. The proportion of 60:40 exotic *versus* local cattle crossbreeds encountered on the 151 studied farms closely agrees with data published by the National Dairy Development Board (NDDB) for Karnataka in 2015 (NDDB 2015). The share of exotic cattle genetics is continuously increasing all over India due to the substantial use of artificial insemination programs (Saha and Bhattacharyya 2020). The number of heifers, dry and lactating cattle per herd was only marginally (1 head) higher than herd size data from 2015 published for rural and urban districts of Bengaluru (NDDB 2015), and compared well to data of a more recent study (Pinto et al. 2020b). The average daily milk yield per lactating cow as reported by the farmers was 2.2 L higher than the production of Indian Sahiwal cows (6.2 L d^−1^) under zero-grazing management (Arora et al. 2022). The high reliance (78%) of dairy farmers on dairy cooperatives for selling milk was similar to the situation in Gujrat and Kerala state. Due to land constraints, almost 30% of the interviewed farmers could not provide sufficient shed space to their animals, a situation also reported from peri-urban dairy farms in Odisha state (Acharya et al. 2022). Such suboptimal housing conditions can directly affect milk yield and cattle health (Pinto et al. 2020b; Arora et al. 2022). The encountered feeding strategies were similar to those reported from other Indian megacities or urban regions, with 24% to 95% of dairy farmers from cities and regions such as Goa, Kerala, Indore, Jaipur, and Nadia feeding concentrates to their cows, especially pregnant and lactating ones (Prajapati et al. 2021). In addition, these authors reported that a majority of farmers were cultivating fodder grasses, preserving different straws, and making silage. The fact that almost two-thirds of the surveyed farmers were unable to provide sufficient fodder for their animals is consistent with reports from South Gujarat and Indore, where 55% to 70% of dairy farmers reported fodder shortages (Rathva et al. 2020; Prajapati et al. 2021). This situation pushes farmers to use naturally growing vegetation from public land resources and food leftovers (Mundoli et al. 2017; Reichenbach et al. 2021a). Significant use of food leftovers and feed resources from public lands was also found in medium-scale dairy systems in Sri Lanka (Weerasinghe 2019). Another challenge in Bengaluru was the high incidence (80%) of drinking water scarcity. A recent study showed that in five Indian megacities including Bengaluru, 33% and 40% of the households have very low and medium levels of access to water for daily needs (Saroj et al. 2020). Scarcity of ground-, lake-, and river-water for domestic, crop, and livestock use has also been noted in Bengaluru metropolitan area due to rapid urban expansion (Unnikirshnan and Nagendra 2018). Although almost all interviewed farmers had vaccinated their cattle against FMD, 40% perceived that their cows were not healthy. The problem of impaired cattle health was also encountered by small-scale dairy farmers in the cities of Karnal, Kolkata, and Guwahati (Sharma et al. 2020). In addition, 10% of the interviewed farmers felt that poor housing conditions and space constraints were responsible for high shed temperatures, which can impair cattle health and provoke heat stress in cows (Pinto et al. 2020b; Velayudhan et al. 2022).

In addition to heat stress problems that are accentuated by lack of drinking water, the quality of forages from uncontrollable sources, namely lake shores and (third parties’) food leftovers, may negatively affect cattle health, as indicated by the results of the logistic regression. From the point of view of proximate constituents, especially the concentrations of CP, NDF, and ADF in alligator weed, the commonly available forage sources compared to high-quality pasture vegetation (Kumar and Vishwakarma 2005; Soder and Muller 2016; Bulyaba and Lenssen 2019). The food leftovers analysed in the present study were also of high quality, with NDF and ADF concentrations lower than in food leftovers fed to Bangladeshi cows (Das et al. 2018). This was due to the fact that the farmers in Bengaluru collected more food waste from restaurants and wet markets with a higher share of cooked and leafy vegetables, which contain high quality NDF and ADF (Lamba et al. 2016; Evan et al. 2020). According to Jagati et al. (2021), cooked food and leafy vegetable leftovers are potential sources of calcium for lactating cows. In the case of water hyacinth, the higher concentrations of Ca might be due to mineral weathering and the presence of rocks and/or rocky soils in water catchment areas (Jagati et al. 2021). Similarly, the higher concentration of P could be due to the high capacity of water hyacinth to absorb and store nutrients (Heard and Winterton 2000).

Unfortunately, the good nutritional quality of Bengaluru’s lakeshore vegetation is compromised by considerable heavy metal contents. However, this has already been reported in several studies on lake macrophytes in Bengaluru (Jumbe and Nandini 2009; Varalakshmi and Ganeshamurthy 2012; Ramachandra et al. 2018, 2020; Hamsa and Prakash 2020) and is also a problem in other megacities such as in Hyderabad along the Musi river (Raj et al. 2006), and in Erode district in Tamil Nadu (Yasotha et al. 2021).

Heavy metals in feed can originate from mining, smelting, burning of fossil fuels and coal, production of glass, steel, batteries, leather or clothing, and can accumulate in various organs of the animal (Guvvala et al. 2020; Wrzecińska et al. 2021). For example, excess dietary As and Cr accumulate in liver, heart, lungs, and kidneys (Dhaliwal and Sushma 2016; Guvvala et al. 2020; Wrzecińska et al. 2021). In addition to the organs mentioned, Cd and Pb can also accumulate in genital organs (Guvvala et al. 2020; Volkov and Ezhkova 2020; Wrzecińska et al. 2021). This results in fertility problems, premature calving, and abortion, but also in hypertension, cancer, nervous and cardiovascular disorders (Alam and Silpa 2020; Guvvala et al. 2020; Wrzecińska et al. 2021).

In the current study, the concentration of heavy metals did not vary between different fodder types, indicating that the respective plants have similar uptake capabilities (Kfle et al. 2020). In contrast, heavy metal concentrations varied significantly between the ten lakes studied, as some of them, especially Narasappanhalli and Bellanduru lakes, are located close to or within industrial areas (MSME 2016; Shankar 2017). A recent official report counted 91,312 small- and medium-size registered factories in Bengaluru (MSME 2016). Most of the waste generated by these factories is not properly treated, resulting in solid factory waste being dumped in unused areas, including lake shores, and liquid waste being discharged directly into drains (Kumar et al. 2009; Gupta et al. 2015; Borthakur and Govind 2017) that often end up in rivers or interconnected lakes. Such practices increase heavy metal concentrations in soil, surface water, and lakeshore vegetation (Kumar and Vishwakarma 2005; Jumbe and Nandini 2009). High concentrations of heavy metals in vegetation used for livestock feeding entail a direct health threat to the animal and, indirectly, to consumers of animal source food (Kumar and Vishwakarma 2005). Although no data is available on the amount of fodder biomass produced near these lakes and used by livestock farmers and cows, respectively, the results of the logit model indicate an increased health risk for cattle fed with lake fodder. Thereby, it should be noted that the aspect of heavy metal contamination of lake fodder was not discussed with the interviewed farmers in order to avoid bias in their assessment of cattle health.

## Conclusions

Dwindling space for housing animals and cultivating forages is a major threat to dairy farmers in and around megacities as a result of rapidly progressing urbanisation. In the case of Bengaluru, there is the additional difficulty of providing sufficient drinking water for the cows. In the hot climate and with high indoor temperatures in narrow barns, this aggravates heat stress and compromises animal health. Feeding cows with food leftovers and lakeshore vegetation are frequently employed strategies to overcome feed shortages. While the nutritional quality of these forages is equivalent to that of high-quality pasture vegetation, elevated concentrations of heavy metals were detected in a majority of samples from lake fodder plants. The results of the logistic regression indicated a negative effect of lake fodder use on cattle health as perceived by their owners, which may indicate (chronic) intoxication of cows with arsenic, cadmium, chromium, or lead. However, further research is needed to quantify the animals’ daily consumption of such hazardous forages and to investigate whether ingested heavy metals enter the human food chain. Independent of such studies, farmers and farm advisors need to address the multifaceted challenges of urbanisation to effectively ensure cattle health.

### Supplementary Information

Below is the link to the electronic supplementary material.

Supplementary file1 (DOCX 14 KB)

## Data Availability

The datasets generated and/or analyzed in the current study are available from the corresponding author for scientific purposes upon written request.
