# Supplementary material for: Urbanisation threats to dairy cattle health: Insights from Greater Bengaluru, India
Source: Trop Anim Health Prod. 2023 Oct 5;55(5):350. doi: 10.1007/s11250-023-03737-7 (PMC10556117; doi:10.1007/s11250-023-03737-7)
Supplement: Supplementary file 1 — Supplementary file1 (DOCX 14 KB) [file 11250_2023_3737_MOESM1_ESM.docx]

**Appendix A**

**Table S1** Description of the variables collected in the interviews with small-scale dairy farmers in Greater Bengaluru, India.

| Variable | Description |
| --- | --- |
| **Socio-economic details of the household** | |
| Household size | Total number of household members |
| Head of household: age, gender, marital status, education, employment | Characteristics of the household head |
| Importance of dairy as an income for the household | Dairy farming is the only income / Dairy farming is the main income amongst others / Dairy farming is a complementary income / Dairy farming is no income source / Don’t know or prefer not to answer |
| **Dairy herd** | |
| Lactating cows | Number of cows currently milked |
| Dry cows | Number of cows currently dry |
| Heifers | Number of heifers, identified as pregnant or already inseminated at least once |
| **Management variables** |  |
| Breed | Dominant cattle genetics in the herd: Local breeds / Exotic breeds / Multiple crosses of exotic x local with a higher share of exotic breeds (= exotic crosses) or local breeds (= local crosses) |
| Night housing | Type of the night resting place (open area / closed area) |
| Shed temperature | Average shed temperature (high / normal) |
| Shed space | Shed space for cows (sufficient / insufficient) |
| Feed supply | Amount of feed supplied to cows (sufficient / insufficient) |
| Concentrate | Daily or very regular supply of concentrate feed (yes / no) |
| Food leftover use | Feeding of leftovers from meals or kitchen waste, wet market waste, industrial fruit peels (yes / no) |
| Lakeshore fodder | Daily or very regular supply of vegetation harvested or grazed on lake shores (yes / no) |
| Drinking water supply | Amount of drinking water supplied to cows (sufficient / insufficient) |
| Walking to pasture | Daily or very regularly walking of cows to a pasture site (yes / no) |
| Vaccination | Vaccination against foot-and-mouth disease (yes / no) |
| Health status | Overall health condition of all cows in the herd (healthy / unhealthy) |
